# Impact of heterotopic ossification following lumbar total disk replacement: a systematic review

**DOI:** 10.1186/s12891-022-05322-9

**Published:** 2022-04-23

**Authors:** Colleen Hood, Reza Zamani, Mohammad Akrami

**Affiliations:** 1 Medical School, College of Medicine and Health, University of Exeter, Exeter, UK; 2 Department of Engineering, University of Exeter, Exeter, UK

**Keywords:** Heterotopic ossification, Lumbar spine, Arthroplasty, Spine surgery, Disc/disk replacement, Degenerative disc/disk, Disc/disk disease, Clinical outcome, Systematic review

## Abstract

**Background context:**

Lumbar total disc replacement (TDR) is an alternative to lumbar fusion in the treatment of lower back pain and reduces the risk of adjacent segment degeneration. Heterotopic ossification (HO) has been identified as a common complication following lumbar TDR.

**Purpose:**

This systematic review aims to determine the prevalence, risk factors and clinical and radiological impact of HO following lumbar TDR.

**Study Design:**

Systematic Review.

**Methods:**

MEDLINE, Scopus, PubMed and Cochrane Central were searched for articles that referred to lumbar TDR and HO. The hits were assessed against inclusion and exclusion criteria. Data from each included study was extracted and analysed with respect to the study aims.

**Results:**

Twenty-six studies were included in this review and the pooled prevalence of HO was estimated to be between 13.2% (participants) and 15.3% (vertebral levels). TDR clinical outcomes were not found to be reduced by HO and there was insufficient data to identify a given impact upon radiological outcomes. Age and follow up time were identified as potential risk factors for HO.

**Conclusions:**

This review was hampered by inconsistencies in the reporting of HO across the studies. We therefore recommend that a set of guidelines should be produced to aid future researchers and reduce the risk of bias.

## Introduction

Lumbar intervertebral disc replacement is an alternative to lumbar fusion in the treatment of symptomatic degenerative disc disease and lower back pain [1–6]. The formation of heterotopic ossification (HO) has been identified as a common complication of lumbar total disc replacement. HO has been identified as a concern following total disc replacement (TDR) as in severe cases it has been shown to hinder the movement at the site the TDR device [7]. In addition, patients displaying severe HO have also been associated with an increased risk of developing adjacent spinal segment degeneration [3]. The impacts of HO following Cervical TDR have been evaluated to a greater extent than HO following lumbar TDR and the majority of these studies have shown that HO does not have a statistically significant impact on the clinical outcomes of the cervical t TDR surgery [5–8]. However, to date there has been no systematic review to investigate the wider impact of HO on the outcomes of TDR. in the lumbar region of the spine. This review aims to determine the clinical relevance and importance of HO so as to determine whether it is a high priority for further research and intervention.

Lower back pain has been shown to be the leading cause of physical disability worldwide; in more economically developed countries, over 70% of the population are affected by lower back pain at some point in their lifetime [9–11]. Lower back pain is frequently indicative of intervertebral disc (IVD) degeneration, a process that results in the composition change and loss of height of the IVD that subsequently disrupts the natural biomechanics of the spinal segment [12–14]. IVD degeneration is estimated to be present in 90% of people aged over 55 years and the prevalence of symptomatic IVD degeneration increases with age. Moreover, with proportional increases in both the global ageing population and the prevalence of symptomatic IVD degeneration there is an urgent need to develop and improve upon existing treatments [12, 15].

Lumbar fusion was once thought to be the gold standard in the treatment of lumbar IVD degeneration that does not respond to non-surgical treatments [16, 17]. However, patients who undergo intervertebral fusion surgery have a greater risk of developing adjacent segment degeneration (ASD) than patients who undergo lumbar TDR and as a result fusion is associated with higher reoperation rates [18–21]. ASD arises due to a lack of mobility at the intervertebral level and disrupts the natural biomechanics leading to a transfer of stress onto the adjacent intervertebral discs that can accelerate their degeneration [2, 18, 20]. The success rate, patient satisfaction and complications rate of lumbar fusion have been shown to be inferior to those of motion preserving devices such as lumbar total disc replacement [4, 18, 19, 21].

Lumbar total disc replacement is an alternative procedure to fusion of the spinal segments in the management of lower back pain. This procedure aims to relieve the back pain whilst maintaining the range of motion at the spinal segments and thereby reduces the risk of adjacent disc degeneration [1, 3, 5, 18, 22, 23]. The development of HO has frequently been reported following lumbar total disc replacement and is defined as the formation of extraskeletal bone within the soft and connective tissues [3, 24–27]. In this review we refer only to acquired HO and not genetic HO. In the case of lumbar TDR, HO is generally considered to form as a result of abnormal tissue repair after the trauma inflicted during the implantation surgery [25, 26]. The severity and development of the HO has also been associated with the severity of the initial trauma [28, 29].

Osteogenic factors such as bone morphogenetic proteins are thought to be required for osteogenesis [26, 30]. Non genetic HO develops through both endochondral and intramembranous ossification processes [27, 31, 32]. Endochondral ossification is defined as the replacement of cartilage with bone and is the process by which bone tissue first forms during foetal development [33]. On the other hand, intramembranous ossification derives from mesenchymal progenitor cells [34]. Meyers et al. [31] propose that HO lesions may develop through a spectrum of endochondral dominant or intramembranous dominant processes whereas sampling of periarticular ossifications revealed that the bone growth following arthroplasty is likely to be entirely endochondral in nature [32]. Foley et al. [32] describe the process of endochondral osteogenesis as starting with perivascular lymphocytic infiltration and migration into soft tissue, proceeded by reactive fibroproliferation and neovascularity [32]. The final stages results in the formation of a cartilage intermediate that is finally replaced by the endochondral bone that presents as heterotopic ossification [32].

Radiographs and computed tomography are the current gold standard techniques used to detect and diagnose HO [25, 26]. However, these techniques often lack the sensitivity to detect HO in the early stages of development [35] The description and classification of HO severity into four classes following total disc replacement has been described by McAfee et al. [36]. Despite this grading system, the clinical impact of HO has been hard to predict from the severity of the bone formations [36]. Complete fusion of the spinal segment and zero degrees of motion is characteristic of Grade IV HO [36]. Despite this, previous studies focusing on the impact of HO on cervical TDR have shown that reduced range of motion (ROM) at the spinal segment is not always indicative of poorer clinical outcomes such as perceived pain and disability index [3, 6, 8]. In contrast, Hui et al. [3] associated severe HO (McAfee grade III and IV) following cervical TDR with an increased risk of developing adjacent segment degeneration. However, in a more recent study by the same authors no association was found between severe HO and biomechanical changes of the cervical spine and therefore these results should be considered with caution [2, 3].

Several risk factors have been associated with HO, although there has been much disparity in the results. Male sex has been associated with significantly increased risk of HO in both cervical spine and hip arthroplasty [1, 7, 37–39]. In addition, a recent study found that male mice formed approximately 30% more HO than female mice and the authors suggest that increased signalling via bone morphogenetic protein and insulin like growth factor-1 pathways in males may explain these findings [40]. Despite this, there is insufficient evidence to determine if male sex is a predisposing factor for HO in humans. Two studies reported a positive and significant association between single level cervical TDR and the development of HO [1, 37]. Yi et al. [37] proposed that the progression of HO is influenced by the biomechanical environment and Hui et al. [1] go further as to suggesting that multilevel TDR is more effective at restoring the natural biomechanical environment than single level TDR and hence the difference in HO rates. Participant age, artificial disc design and studies with longer follow up durations are also factors that have been associated with an increased risk of developing HO [1, 6, 7, 37, 41].

The aim of this systematic review is to determine the clinical and radiological relevance and importance of heterotopic ossification following lumbar intervertebral disc replacement. This will be achieved by completing the following objectives: (I) calculating the pooled prevalence of HO across all available studies following lumbar TDR. (II) Calculating the mean percentage change in clinical and radiological outcomes and establishing any impacts of HO on the clinical and radiological outcomes of lumbar TDR and (III) Evaluating the risk factors for HO.

## Methods

### Search strategy

**Fig. 1:**
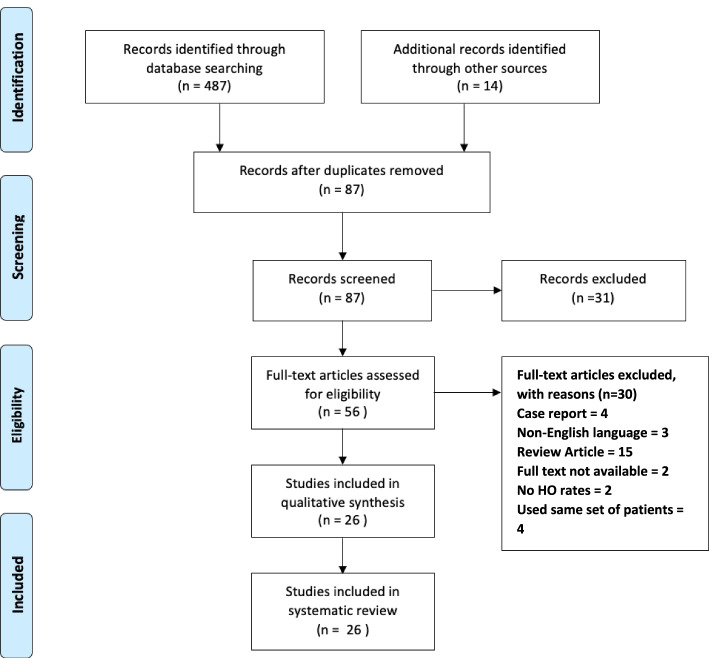
PRISMA flow diagram

A systematic review of literature was conducted in accordance with the guidelines for Systematic reviews and meta-analyses in spine surgery and the Preferred Reporting Items for Systematic Review and Meta-Analysis protocols (PRISMA-P) [42, 43]. Multiple databases (MEDLINE, Scopus, PubMed and Cochrane Central) were searched using the following key terms: “heterotopic ossification,” “heterotopic,” “bone,” “lumbar,” “arthroplasty,” “disk/disc replacement,” “disk/disc,” “prosthesis,” and “degenerative disk/disc disease.” The combination of terms used in each search are shown in Table 1 and an overview of the search process in Fig. 1.

**Table 1:** Search term combination and total hits from the systematic search of MEDLINE, Scopus, PubMed and Cochrane Central databases

Search combination and total hits	
Search Combination	Total Hits
“Heterotopic ossification” AND lumbar AND arthroplasty	89
“Heterotopic ossification” AND lumbar AND “disc replacement OR disk replacement”	94
“Heterotopic ossification” AND lumbar AND “disc OR disk” AND prosthesis	80
Heterotopic AND bone AND lumbar AND arthroplasty	55
Heterotopic AND bone AND lumbar AND “disc replacement OR disk replacement”	47
Heterotopic AND bone AND lumbar AND “disc OR disk” AND prosthesis	44
“Heterotopic ossification” AND lumbar AND “degenerative disc disease OR degenerative disk disease”	54
“Heterotopic ossification” AND lumbar AND “degenerative disc disease OR degenerative disk disease”	24

### Article selection

Literature was considered up to the publish date of February 2021 and ranged back to 1996. At the beginning of the search no cut-off date was chosen. However, after reviewing the results, 1996 was chosen as the final cut of date as it was the earliest search hit within 25 years of the final search date. The reference lists of the selected articles were reviewed for potential studies. Article duplicates were removed, and the titles and abstracts of the remaining articles were screened. The full texts were then reviewed using the following inclusion and exclusion criteria. For multiple published articles that included the same study population the latest published article was included in this review.

**The Inclusion Criteria Used:**

- Studies concerning lumbar TDR that reported either HO patient rates or HO operative segment rates
- Randomized, non-randomised, prospective and retrospective studies
- Study subjects aged 18 years and over

**The Exclusion Criteria Used:**

- Literature reviews, case reports, and conference reports
- HO rates not reported
- Non-English texts without translation
- TDR in the cervical spine
- Duplications of publications
- Study follow up period of less than a year

### Data extraction

After study selection, data was extracted from each of the studies and recorded in a table. The data extracted included: study design, year of publication, sample size, mean participant age, follow up period, type of prosthesis, spinal level of surgery, HO rate. Two clinical outcomes were extracted and were as follows: visual analogue scores (VAS) for participants’ perceived pain and Oswestry disability index (ODI) scores. For the radiological outcomes, ROM at the index level was extracted. Patient demographics and surgery details were also extracted and recorded in tables for the analysis of potential risk factors of HO.

### Study quality assessment

The methodological quality and risk of bias of the randomised controlled trials was assessed by using the checklist published in the updated guideline for systematic reviews by the Cochrane Back and Neck Group [44].The risk of bias for the non-randomised studies were assessed using the 12-point scale of the Methodological Index for Non-Randomised Studies (MINORS) for non-comparative studies [45]. Journal strength was also assessed through SCImago Journal ratings [46].

### Data analysis

An estimation of the pooled prevalence of HO was calculated by dividing the total number of participants/levels affected by HO by the total number of participants/levels across the 26 studies. Mean percentage changes in clinical and radiological outcomes were calculated for each study. Pearson correlation coefficients were calculated, and a regression analysis was conducted to determine the significance of the correlation between percentage of participants/index levels with HO and the mean percentage change of the outcomes. Regression analysis was also applied to both patient demographics and surgery details and the proportion of participant/index levels with the rate of HO per study to identify any population risk factors.

## Results

### Search results

487 studies were identified from the initial database search and a further 14 articles found by searching the reference lists of the included studies. 414 articles were duplications and subsequently removed. The titles and abstracts of the remaining 87 articles were screened and 31 were excluded.

The full text of the remaining 56 articles were screened against the inclusion and exclusion criteria. A total of 30 articles were removed and the reasons for exclusion can be seen in Fig. 1. The remaining 26 studies were included in this systematic review.

### Study characteristics

Out of the 26 studies, 5 are randomised control trials (RCT’s), [47–51]; fifteen studies in this review are non-randomised prospective, [52–66] and six studies are retrospective [67–72]. The publication date of the studies ranged from 1996 to 2019. The mean number of participants across all the studies was 95 and cohort size ranged from 15 to 405. The total number of participants included in this review across the 26 studies is 2269 (including drop outs.) All 26 studies reported the rate of HO in the study population, of which nine reported in terms of participants, six in terms of spinal level and 11 in both. Half of the studies [13] reported on the different McAfee grades of HO. In three studies, HO was reported only if it interfered with the ROM at the index [49, 54, 62]. In addition, none of the 26 studies reported the use of HO prophylaxis techniques. An overview of the study characteristics and outcome measures are shown in Table 2.

**Table 2:** Study characteristics

Ref	First Author	Study Location	Year of Publication	Journal	Study Design	Sample Size	Outcome Measure(s)	Mean Follow Up (years)
[52]	G.Pokorny	Brasil	2019	World Neurosurgery	Non random, prospective	60	Heterotopic ossification rates, Pain VAS scores, ODI scores, reoperation rates	7.75
[47]	F. Gornet	USA	2019	Journal of Neurological spine	RCT	577	Heterotopic ossification rates, Pain VAS scores, ODI scores, ROM, Reoperation rates	5
[67]	H.Park	Korea	2018	The Spine Journal	Retrospective case review	65	Heterotopic ossification rates	8.7
[68]	S.Lu	China	2018	The Spine Journal	Retrospective	35	Heterotopic ossification rates, Pain VAS scores, ODI scores	15.2
[53]	V,A. Byvaltsev	Russia	2017	Coluna/Columna	Non random, prospective	156	Heterotopic ossification rates, Pain VAS scores, ODI scores, ROM	3
[54]	A,G. Tohmeh	USA	2015	European Spine Journal	Non random, prospective	64	Heterotopic ossification rates, Pain VAS scores, ODI scores, Reoperation rates	3
[56]	S. Lu	China	2015	European Spine Journal	Non random, prospective	35	Heterotopic ossification rates, Pain VAS scores, ODI scores, ROM, Reoperation rates	11.8
[55]	S.Lu	China	2015	Journal of Spinal Disorders	Non random, prospective	32	Heterotopic ossification rates, Pain VAS scores, ODI scores, ROM, Reoperation rates	2.4
[57]	J,R. Baldeston	USA	2014	Spine	Non random, prospective	15	Heterotopic ossification, ODI scores	9.6
[58]	A,R. Meir	Australia	2013	The Spine Journal	Non random, prospective	28	Heterotopic ossification, Reoperation rates	9.6
[59]	L. Marchi	Brazil	2012	International Journal of Spine Surgery	Non random, prospective	36	Heterotopic ossification rates, Pain VAS scores, ODI scores, Reoperation rates	3
[69]	C. Jones	Australia	2012	Orthopaedic Surgery	Retrospective	25	Heterotopic ossification	2.83
[60]	S. Park	Korea	2011	International Orthopaedics	Non random, Prospective	65	Heterotopic ossification	3.75
[61]	G. Cinotti	Italy	1996	Spine	Non random, Prospective	46	Heterotopic ossification, Reoperation rates	3.2
[70]	M. Putzier	Germany	2006	European Spine Journal	Retrospective	71	Heterotopic ossification, Reoperation rates	17.3
[48]	R. Guyer	USA	2016	Spine	RCT	394	Heterotopic ossification rates, Pain VAS scores, ODI scores, ROM, Reoperation rates	5
[62]	J, P. Lemaire	France	2005	Journal of Spinal Disorders	Non random, prospective	107	Heterotopic ossification	11.3
[49]	R. Guyer	USA	2009	The Spine Journal	RCT	133	Heterotopic ossification rates, Pain VAS scores, ODI scores	5
[50]	R. Garcia Jr	USA	2015	Spine	RCT	324	Heterotopic ossification rates, Pain VAS scores, ODI scores, Reoperation rates	2
[63]	M. Katsimihas	Canada	2010	Canadian Journal of Surgery	Non random, prospective	64	Heterotopic ossification rates, Pain VAS scores, ODI scores, Reoperation rates	4.58
[64]	J,C. Le Huec	France	2005	Orthopaedic Clinics of North America	Non random, prospective	64	Heterotopic ossification rates, Pain VAS scores, ODI scores	2
[71]	A,V. Ooij	Netherlands	2003	Journal of Spinal Disorders	Retrospective	27	Heterotopic ossification	7.58
[66]	E. Van De Kelft	Belgium	2012	World Neurosurgery	Non random, prospective	50	Heterotopic ossification rates, Pain VAS scores, ODI scores	4
[72]	T. David	France	2007	Spine	Retrospective	108	Heterotopic ossification, Reoperation rates	13.2
[51]	P. McAfee	USA	2003	Journal of Spinal Disorders and techniques	RCT	60	Heterotopic ossification	3
[65]	R. Fraser	Australia	2004	The Spine Journal	Non random, prospective	28	Heterotopic ossification rates, ODI scores, Reoperation rates	2

### Methodological quality

**Fig. 2:**
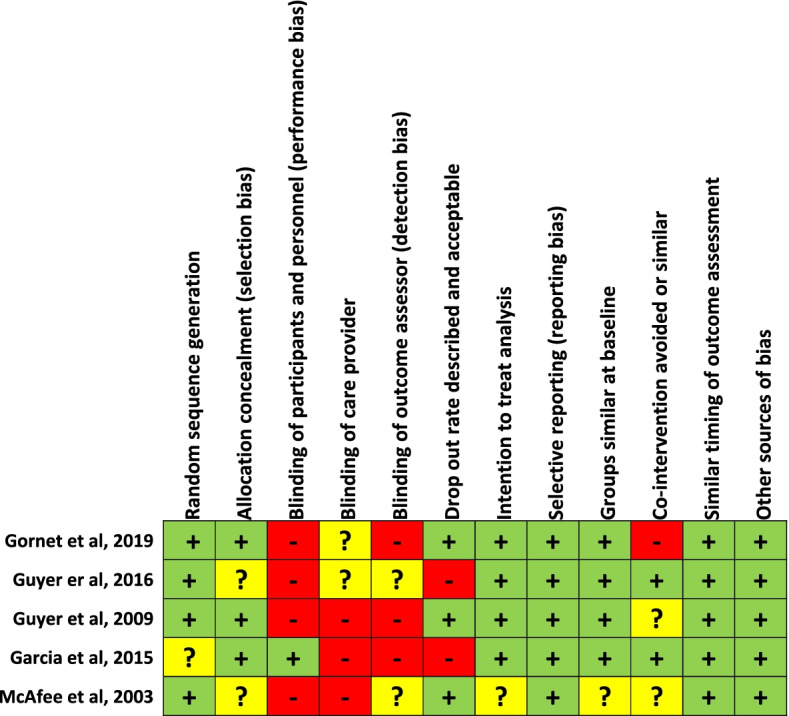
Risk of bias for randomised controlled trials summary figure

15 papers were published in Q1 SCImago rated journals, five from Q2 and one from Q3 and Q4 rated journals [46]. One paper was from a journal that lacked data to rank [46]. The Quality of the five RCT’s was assessed in accordance with the Cochrane Back and Neck Group Guidelines [44]. Overall the risk of bias was low for most of the criteria although a potential bias in the blinding of the, care provider and assessors participants was identified in most of the studies. A summary of the risk of bias for the RCT’s can be seen in Fig. 2. Methodological quality for the non-randomised studies was assessed using MINORS and the mean score was 8.7 out of 12 [45]. Nearly all the studies reported inadequate drop out rates (more than 5%) and all failed to report a prospective calculation of the study size. A summary of the methodological quality of each study can be seen in Table 3.

**Table 3:** A summary of the methodological quality of the studies included in this systematic review

Ref	Study design	Sample size	Drop Out /Withdrawal Rate	Scimago Journal rating	MINORS score (out of 16)
[52]	Non randomised prospective single centre study	60	9—no reasons given	Q2	10
[47]	RCT	577	8 unrelated deaths, 146—no reasons given	Q1	N/A
[67]	Retrospective case review	65	17—no reasons given	Q1	N/A
[68]	Retrospective single centre clinical trial	35	5—no reasons given	Q1	N/A
[53]	Non randomised prospective multi centre study	156	N/A	Q4	8
[54]	Non randomised prospective multi centre clinical study	64	4—no reasons given	Q1	11
[56]	Non randomised prospective clinical trial	35	1 lost to follow up, 1 unrelated death, 1 declined participation	Q1	10
[55]	Non randomised prospective clinical trial	32	2—no reasons given	N/A	11
[57]	Non randmoized prospective clinical data analysis	15	2 lost due to change in contact details	Q1	10
[58]	Non randomised prospective clinical trial	28	2 lost due to change in contact details	Q1	9
[59]	Non randomised prospective single centre study	36	N/A	Q2	7
[69]	Retrospective study	25	3—no reasons given	Q3	N/A
[60]	Non randomised Prospective study	65	N/A	Q1	6
[61]	Non randomised prospective study	46	N/A	Q1	6
[70]	Retrospective	71	18—no reasons given	Q1	N/A
[48]	RCT	394	124—no reasons given	Q1	N/A
[62]	Non randomised prospective study	107	6 lost due to change in contact details, 1 unrelated death	N/A	6
[49]	RCT	133	11 declined participation, 10 early discontinuation and 96 no reasons given	Q1	N/A
[50]	RCT	324	58—no reasons given	Q1	N/A
[63]	Non randomised prospective study	64	7—no reasons given	Q2	11
[64]	Non randomised prospective study	64	No drop outs	Q1	10
[71]	Retrospective	27	N/A	N/A	N/A
[66]	Non randomised prospective study	50	5- lost due to change in contact details	Q2	9
[72]	Retrospective	108	2-unrelated death	Q1	N/A
[51]	RCT	60	No drop outs	Q1	N/A
[65]	Non randomised prospective study	28	N/A	Q2	7

^*^MINORS Methodological Index for Non-Randomized Studies

### Prevalence of heterotopic ossification

HO prevalence varied from 0 to 91% (SD = 30.9) across all 26 studies. Eleven studies reported the rate of HO in terms of participants and levels, nine reported in participants and six reported the rate of HO in the vertebral levels across the patients. Across all 20 studies that reported HO in the participants the pooled prevalence was 13.2% (254/1917). Across the 17 studies that reported HO of the vertebral levels the pooled prevalence was 15.3% (220/1435). The mean prevalence of HO across all 20 studies that reported HO in terms of participants was calculated to be 12.9%. Details of each study’s HO rates and outcomes are shown in Table 4.

**Table 4:** Outcome measures

Ref	HO at the surgical level	Patients with indications of HO at index level	Percentage of patients with HO	HO graded by McAfee or other scale?	Mean percentage change in visual analogue scores for perceived patient pain	Mean percentage change in Oswestry disability scores of patients	Mean percentage change in range of motion at the index level of the artificial disc(s)
[52]	N/A	41	91.1	YES	61.1	60.1	N/A
[47]	11	11	5.88	NO	73.6	66.4	31.8
[67]	30	N/A	50	YES	N/A	N/A	N/A
[68]	26	N/A	74.3	YES	81.6	89.9	N/A
[53]	37	37	23.7	YES	82.6	68.9	10.6
[54]	3	3	5.36	NO	68.5	64.7	N/A
[56]	25	N/A	71.4	YES	82.8	68.1	-37
[55]	N/A	1	3.33	NO	82.6	62.8	27.99
[57]	0	0	0	YES	N/A	81.5	N/A
[58]	12	N/A	85.7	NO	N/A	N/A	N/A
[59]	N/A	7	20	NO	74.4	69.3	N/A
[69]	19	N/A	83	YES	N/A	N/A	N/A
[60]	25	N/A	30.5	YES	N/A	N/A	N/A
[61]	N/A	7	15.2	NO	N/A	N/A	N/A
[70]	N/A	39	73.6	YES	N/A	N/A	N/A
[48]	N/A	32,36	15.9,19.3	NO	69.6, 71.8	63.7, 66.1	15.3
[62]	N/A	3	3	NO	N/A	N/A	N/A
[49]	17	17	18.9	NO	55.7	49.4	N/A
[50]	3,1	3,1	1.6, 1.1	NO	74,68	67, 61	N/A
[63]	0	0	0	yes	50	50	N/A
[64]	3	3	4.69	YES	57.9	47.3	N/A
[71]	N/A	4	14.2	NO	N/A	N/A	N/A
[66]	0	0	0	YES	64.9	69.3	N/A
[72]	7	7	6.6	NO	N/A	N/A	N/A
[51]	1	1	2.22	YES	N/A	N/A	N/A
[65]	N/A	1	3.56	NO	N/A	30.2	N/A

*HO* Heterotopic ossification, *VAS* Visual Analogue Scores for Perceived Pain, *ODI* Oswestry Disability Index, *ROM* Range Of Motion at index level

### Perceived pain visual analogue scores

Thirteen studies reported on the participants mean perceived pain before and after lumbar TDR [47–49, 52–56, 59, 63, 64, 66, 68]. Percentage change in VAS scores before and at the final follow up was calculated and a regression performed against HO. There was found to be no significant correlation between mean percentage change in VAS score and the proportion of patients in the study with HO (*P* < 0.34 at 95% CI). Percentage change in VAS ranged from 50% to 82.8% and the mean improvement across the studies was 70%. Three studies found no significant differences between the mean VAS score and the four McAfee Classes of HO [56, 60, 67]. Jones et al. [69] found a statistically significant improvement of mean VAS pain score for participants with HO (McAfee grades I-III) compared to the group without HO.

### Oswestry disability index

ODI, is a measure of permanent lower back function and disability range from 0 to 100%, where 0% indicates the patient can cope with day-to-day activities with minimal treatment and 100% indicates the patients are bed bound. Sixteen studies reported the participants mean ODI scores before and after lumbar TDR [47–50, 52–54, 56, 57, 59, 63–66, 68]. Percentage change of ODI scores before and at the final follow up were calculated and a regression was performed against HO. There was found to be no statistically significant correlation between percentage change in ODI score and the proportion of patients in the study with HO (*P* < 0.21 at 95% CI). Percentage change in ODI ranged from 30.2% to 89.9% and a mean improvement of 63.1% across the 16 studies. There were no significant differences in improvement of mean ODI scores between the different grades of HO [56, 60, 67].

### Range of motion

Five studies reported on ROM at the index level before and after lumbar TDR surgery and therefore a regression was not performed due to too few publications reporting ROM [47, 48, 53, 55, 56]. The mean percentage change in ROM before and after lumbar TDR ranged from -37% to 28% and the mean across the studies was 8.15% improvement. All five studies reported that patients with HO limiting ROM did not have significantly reduced clinical outcomes compared with participants without HO.

### Age

**Fig. 3:**
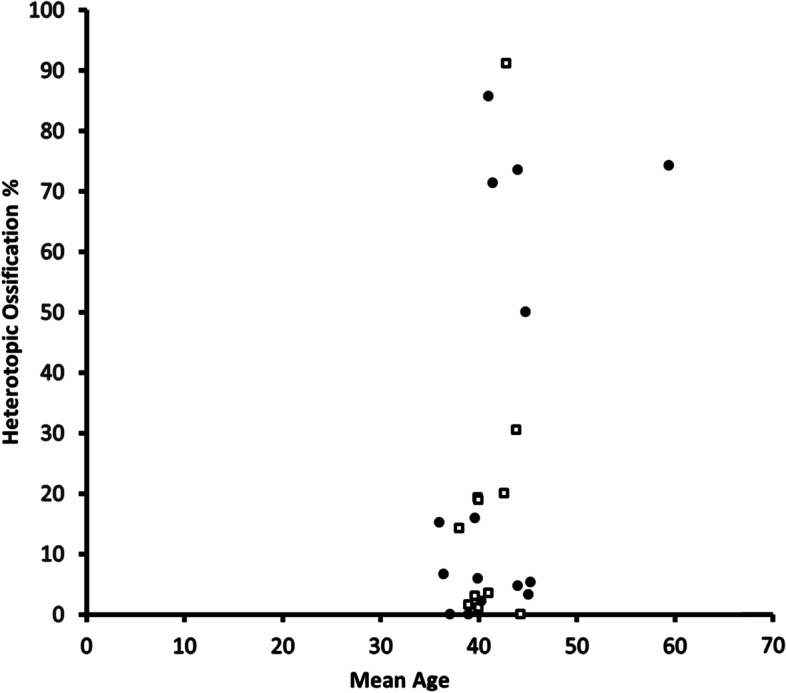
Scatter graph of HO rate and mean age

All studies apart from two reported the mean age of the participants [53, 69]. The mean age ranged from 36 to 59.4 years and the mean across all the studies was 41.7 years. Age and additional patient demographics from each study are shown in Table 5. Regression analysis was performed, and a statistically significant (*P* < 0.3 at 95%CI) positive correlation was found between the mean age of the participants and the proportion of participants with HO as shown in Fig. 3.

**Table 5:** Patient demographics

						Surgical Level/Segment				
Reference	Male (%)	Mean Age	Device design	Smokers(%)	Surgery	L1-2	L2-3	L3-4	L4-5	L5-S1
[52]	52%	42.8	XL-TDR	N/A	Lateral Approach	N/A	3	10	42	N/A
[47]	50%	39.9	Maverick	28.9	Anterior Approach	N/A	N/A	N/A	N/A	N/A
[67]	33%	44.8	ProDisc II (92%), Charite 8%	N/A	Anterior Approach	N/A	N/A	N/A	N/A	N/A
[68]	53%	59.4	Charite III	N/A	Anterior Approach	N/A	N/A	1	18	6
[53]	N/A	N/A	M6-L	N/A	N/A	N/A	N/A	10	43	103
[54]	58%	45.3	XL-TDR	20.3	Lateral Approach	2	3	11	48	N/A
[56]	44%	41.4	Charite III	N/A	Anterior Approach	N/A	N/A	N/A	N/A	N/A
[55]	60%	45.1	Active L	N/A	Anterior Approach	N/A	N/A	3	23	10
[57]	31%	44.3	ProDisc L	N/A	N/A	N/A	N/A	N/A	N/A	N/A
[58]	50%	41	AcroFlex	39.2	Anterior RPA	N/A	N/A	N/A	N/A	N/A
[59]	44%	42.6	XL-TDR	N/A	Lateral Approach	N/A	N/A	4	17	N/A
[69]	48%	N/A	Charite III	N/A	Anterior Approach	1	1	3	9	10
[60]	37%	43.8	Prodisc (91%) and Charite (9%)	N/A	Anterior Approach	N/A	N/A	3	46	33
[61]	46%	36	Charite III	N/A	Anterior Approach	N/A	N/A	4	26	30
[70]	38%	44	Charite I (25%), II(40%), III(35%)	N/A	Anterior Approach	N/A	N/A	2	35	26
[48]	47%	39.6, 39.9	Kineflex (52%) Charite III (48%) Control	N/A	Anterior Approach	N/A	N/A	N/A	46, 48	158, 142
[62]	41%	39.6	Charite III	N/A	Anterior Approach	N/A	N/A	6	69	72
[49]	52%	40	ChariteIII (67%) AND BAk fusion (33%)	N/A	Anterior Approach	N/A	N/A	N/A	26	64
[50]	52%	39,40	Active L (67%), Prodisc OR charite (33%)	39.2	Anterior Approach	N/A	N/A	N/A	62,34	156,72
[63]	39%	39	Charite III	61.4	Anterior Approach	N/A	N/A	N/A	4	53
[64]	39%	44	Maverick	29.7	Anterior Approach	N/A	N/A	2	27	35
[71]	44%	38	Charite III	N/A	Anterior Approach	N/A	1	2	19	11
[66]	48%	37.1	Maverick	N/A	Anterior Approach	N/A	1	2	19	28
[72]	42%	36.4	Charite III	N/A	Anterior Approach	N/A	N/A	1	25	82
[51]	50%	40.3	Charite III (68%), BAK Fusion (32%)	N/A	Anterior Approach	N/A	N/A	N/A	19	41
[65]	50%	41	Acroflex	39.2	Anterior Approach	N/A	N/AA	N/A	9	23

^*^N/A data not reported/unavailable

### Post operation follow up time period

**Fig. 4:**
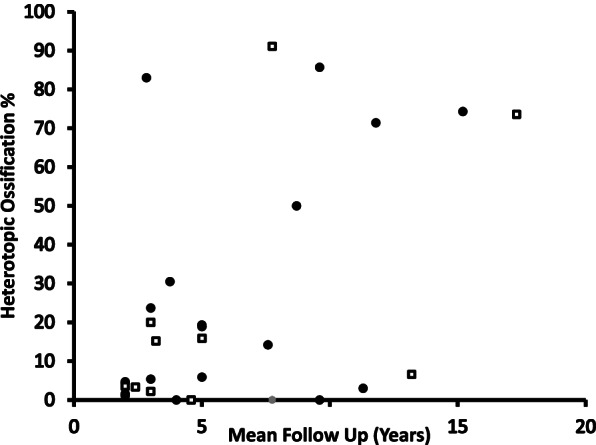
Scatter graph of HO rate and mean follow up

The follow up time ranged from two years to 17.3 years and the mean across all the studies was 6.17 years. After running regression, a statistically significant (*P* = 0.01 at 95%CI) and positive correlation was found between the follow up time period and the proportion of participants with HO as shown in Fig. 4.

### Patient gender index

All studies except one reported the proportion of male and female participants [47, 53].

The range of male participants ranged from 30.8% to 60% and the mean across all studies was 46%. No statistically significant relationship was found between the percentage of male participants and the proportion of participants in the study with HO (*P* = 0.24 at 95%CI).

### Mode of surgical operation and surgical and hospital details

All studies except three reported the surgical approach during the implantation of the artificial disc [53, 57, 67]. Lateral retroperitoneal approach was conducted in three studies [52, 54, 59]. The remainder of the studies reported taking an anterior retroperitoneal surgical approach to implantation of the artificial disc.

21 studies reported the spinal level(s) in which a prosthetic disc was implanted**.** Of which 60% used implants at levels L5-S1 spinal region, 36% at L4-5, 3% at L3-4, 0.4% at L2-3 and 0.1% at L1-2. There was no statistically significant correlation between the regression of the percentage of prosthesis implanted at each level in each study and the proportion of participants with HO (*p* > 0.01). 11 studies reported the mean surgical time during the implant surgery[47–49, 52, 53, 55, 58–60, 65, 72]. The mean surgical time across all the studies was 116 min and ranged from 90 to 168 min. Blood loss during the implant surgery was reported by 10 studies [47–50, 52, 53, 55, 58, 59, 65]. The mean blood loss across all the studies was 169 ml and ranged from 58 to 472 ml. A total of 10 studies reported the mean hospital stay following the implant surgery [47–50, 53, 55, 58, 59, 64, 65]. The mean hospital stay across all the studies was four days and ranged from one to just over eight days. No statistically significant correlation was found between the regressions of the mean surgical time (*p* > 0.7), mean blood loss (*p* > 0.3) or mean hospital stay (*P* > 0.3) and the proportion of patients with HO.

### Artificial disc materials

Ten different types of prosthetic devices were used across all the studies, of which four were metal-on-metal in design (XL-TDR, Maverick and Kineflex) and the rest metal-on-plastic in design. Metal-on-metal discs were implanted in seven out of the 26 studies[47, 48, 52, 54, 59, 64, 66]. A Mann–Whitney U test was performed and showed that the percentage of participants with HO was not statistically significantly (*P* > 0.9 at 95% CI) different between studies with metal-on-metal prosthesis and metal-on-plastic implant designs.

## Discussion

This systematic review aimed to establish the clinical relevance and impact of heterotopic ossification on the patient’s quality of life following lumbar intervertebral disc replacement. At the time of writing this report, this is the first systematic review looking at HO following lumbar TDR and to estimate the prevalence of HO in this spinal region. A total of 26 studies were found eligible for inclusion and composed of RCT’S, non-randomised clinical trials and retrospective study designs. Heterotopic ossification was found to be prevalent in 15.3% (220/1435) of the spinal levels and 13.2% (254/1917) of participants. The discrepancy between these values could be explained by inconsistent reporting of HO across the studies with only 17 studies reporting the spinal levels with indications of HO and 20 reporting in patients. In previous systematic reviews that aimed to establish the prevalence of HO following cervical TDR all studies expressing HO in terms of patients were excluded [1, 2]. In this review however, a limited number of available studies called for less stringent exclusion criteria and this identifies a need for the development of standardised reporting guidelines for expressing HO and possibly other spinal disorders.

Two recent systematic reviews and meta-analysis by Hui et al. [1, 2] estimated the prevalence of HO following cervical TDR to be 29.1% and 32.5%. Similarly, Kong et al. [6] estimated HO prevalence following cervical TDR to be 38%. The discrepancy between the present study and these reports could be due to several factors. This review focused on HO following lumbar TDR and the prevalence may differ from the prevalence of HO following cervical TDR. Secondly, no meta-analysis was conducted and therefore the simple estimation was derived by dividing the number of participants/levels affected by HO by the total number of participants/levels across all the studies. Moreover, only 26 studies met the inclusion criteria for this review whereas Hui et al. [1] included 94 in their study. This may have contributed to the lower value of estimated prevalence in this review due to a smaller pooled population sample. Lastly, three of the included studies seen in this review reported the rate of HO to be zero whereas Kong et al. [6] excluded these studies in their systematic review. Lastly, it is probable that the prevalence of HO will vary between the lumbar and cervical regions of the spine due to differences in the kinematics, weight distribution and anatomy between the two regions.

The rate of HO varied greatly across the studies included in this review. Three studies reported zero cases of HO, whereas six studies found evidence of HO in over 70% of the study population [52, 56–58, 63, 66, 68–70]. This variation may be explained by the lack of consistency in detection and diagnosis of HO. HO was the primary concern in some of the studies reviewed, whereas in others it was a secondary outcome. In studies where HO was the primary outcome, meticulous searching for indications of HO may have resulted in elevated HO detection rates. In addition, the variation in sample size from 15 up to 405 participants may explain the differences in HO rates across the studies. In general studies with fewer participants were found to have higher prevalence of HO than studies with a greater sample size. Other factors for the disparity in HO rates between the current study and other reports include differences in participant inclusion and exclusion criteria, surgical approach and technique, and collective participant demographics such as ethnicity, and reason for lower back pain.

This study found no significant correlation between the rate of HO and the mean percentage improvement in ODI and VAS pain scores. The studies that reported the mean change in ODI and VAS pain scores, all saw an overall improvement at last follow up despite reports of high rates of HO in some studies. These findings seem to indicate that, in general, HO does not significantly affect the clinical outcomes of lumbar TDR. This is also supported by Chen et al. [5]. These results need to be interpreted with caution however, as there was an absence of data for the changes in both ODI and VAS for each McAfee grade of HO in all but four studies [56, 60, 67, 69]. Three of these studies found no significant differences in mean improvement of VAS and ODI between the grades of HO [56, 60, 67]. Jones et al. [69] however, reported a statistically significant improvement of VAS pain scores in groups with McAfee grades I, II and III HO compared to groups without HO. These findings are somewhat limited as the preoperative pain scores were obtained retrospectively due to a lack of baseline data and therefore should be considered with caution. Overall, the studies in this review seem to agree that McAfee grades one and two HO do not impact the clinical outcomes of HO to a statistically significant degree.

Five studies reported the mean change in ROM before and after Lumbar TDR and all concluded that in general, patients with HO limiting ROM did not have significantly reduced clinical outcomes than participants without HO. In addition, four studies suggested that reduced range of motion was typical in spinal segments with McAfee HO grades III or IV [56, 60, 67, 68]. Pokorny et al. [52] found that although 92% of the participants had signs of HO, 82% still maintained some range of motion at the index spinal segment and again did not affect either the ODI or VAS pain scores. Lu et al. [56] were the only authors to report a reduction in mean postoperative ROM compared to preoperative values. The authors suggest this decrease in ROM may have been resulted from hindrance in soft tissue changes and also imply a mental component where the patients develop an aversion to movement due to pain [56].

In this study, a weak but positive association between participant age and the development of HO was identified. These results are consistent with the findings of a clinical trial published in 2005 [7]. In contrast, a more recent review and meta-analysis by Hui et al. [1] found no evidence to suggest that older age is associated with HO; the authors did find a relationship between both follow up time and male sex and greater rates of HO (McAfee grade III and IV). This current study also found a positive relationship between follow up time and the rate of HO and therefore suggests that increased implant time in the body may increase the risk of developing HO. This assumption should be made with caution though as Kong et al. [6] found that HO prevalence increased only in the short and mid-term follow up. Although the prevalence of HO did not increase in the long-term, pre-existing HO did continue to develop into severe HO suggesting that HO may get progressively more severe with time [6].

Regarding surgical procedures, three studies described a lateral approach during the implant surgery, while the remaining studies implanted the artificial disc via the typical anterior retroperitoneal approach [52, 54, 59]. The anterior approach is thought to be more invasive and has a higher risk of adverse events than the lateral approach [52, 54, 59, 73]. Pokorny et al. [52] presented the highest rate of HO out of all the studies included in this review. The authors attributed this to the lateral surgical approach where incomplete removal of the contralateral annulus tissue could have acted as a scaffold for HO bone growth [52]. Interestingly all three studies that implanted via the lateral approach note that HO developed primarily on the contralateral aspect of the disc, whereas all the other studies report that HO was detected on the anterior side. This suggests that the approach may have an impact on the location of the HO and supports the theory that HO develops as a response to trauma inflicted during the implantation surgery. Moreover, Lemaire et al. [62] found that lateral HO tended to lead to fusion whereas the index spinal level maintained motion when the HO was located anteriorly. Overall, the impact of surgical approach on the severity of HO has yet to be established and is likely to be an important area of research to determine the clinical importance of HO in the future.

The methodological quality of the studies is almost certain to have affected the results of this review. Three studies reported only ROM limiting HO and this potentially increased the risk of outcome reporting bias [49, 50, 54]. Ideally, all indications of HO should have been reported and the grades identified. In addition, many of the included studies failed to provide critical patient information and outcomes that are essential for determining the clinical importance of HO and identifying potential risk factors. McAfee et al. [51] failed to distinguish between groups of participants who underwent lumbar TDR and BAK interbody fusion when reporting demographics and clinical outcomes and instead, reported combined data for the two groups and consequently severely limited the impact of their study [51].

This systematic review has some important limitations to consider. Firstly, owing to the limited number of available studies, articles that expressed HO in participants were included. This resulted in difficulty when estimating the prevalence of HO, as some of the studies reported in levels and others in participants. This also called in to question the quality of such studies, as in some participants who had multi- level TDR surgery it was often ambiguous how many of the implants were affected by the HO. Secondly, even with the broad inclusion criteria the number of studies included in this review, the number of studies is still relatively small and is only representative of 12 countries across the globe and therefore may not be representative of all patients who undergo lumbar TDR in the wider population.

The methods used by the included studies to detect HO included radiography, magnetic resonance imaging and CT scans. This inconsistency amongst the studies may have introduced error into the estimation of pooled prevalence of HO. For example, Lemaire et al. [62] noted that in anteroposterior and lateral radiographs, only one case of HO was detected. However, with the use of computer tomography indications of HO were found in the majority of spinal segments. In addition, Park et al. [67] recognise that their use of anteroposterior radiographs to detect HO may have resulted in reduced HO numbers, as lateral ossification is difficult to detect using anteroposterior radiographs.

## Concluding remarks and recommendations

This is the first systematic review to focus on heterotopic ossification following TDR in the lumbar region of the spine. The findings from this review suggest that mild HO (McAfee grades I-II) may not impact the clinical outcomes of lumbar TDR and supports previous systematic review and meta-analysis for HO formation after cervical TDR. However, there is currently not enough information to determine the clinical impact of grade severe HO (McAfee grades III-IV). In regard to radiological outcomes, more severe HO has been shown to decrease the ROM of the index spinal segment. However, there has been no clear evidence to suggest that decreased ROM results in poorer clinical outcomes. Age and follow up time after implantation of the artificial disc were associated with higher HO rates, both of which have previously been recognised as potential risk factors of HO following cervical TDR [1, 6].

The major limitations with this systematic review stem from lack of consistency across the studies when detecting and reporting the rate and grade of heterotopic ossification. An approach to solve this problem could be to produce a set of guidelines to aid in the reporting of HO. These guidelines could help to standardise the method of diagnosis and reporting of HO and may help to reduce the risk of bias when comparing and pooling data. The aforementioned guidelines could include the following terms: I) Heterotopic ossification should be diagnosed using the current gold standard (currently radiographs and computed tomography) and any abnormal findings should be investigated further with a second imaging approach. II) Heterotopic ossification should always be reported in terms of spinal segments/ levels and not the patients. III) Heterotopic ossification should always be graded by McAfee classification or other suitable alternative, or if a grade is not suitable to describe the ossifications, a detailed description should be given. IV) Participant demographics and outcomes should be reported for each grade of HO. The latter point could provide crucial information and insight into the clinical impact of severe (grade III and IV) HO, and a research question that is still yet to be answered. It is worth noting that these guidelines are an ideal, and it is unlikely that all hospitals and treatment centres globally could be standardised to such an extent. The findings from this systematic review may help to understand the impact of HO on the clinical outcomes of lumbar total disc replacement. Moreover, it identifies the need for the standardisation of future reporting of HO and the need for further meta-analysis on the prevalence and clinical impact of severe HO.

## Data Availability

All data generated and analysed during this study are included in this published article.

## Abbreviations

ASD: Adjacent segment degeneration
HO: Heterotopic ossification
IVD: Intervertebral disc
MINORS: Methodological index for non-randomized studies
ODI: Oswestry disability index
PRISMA-P: Preferred reporting items for systematic reviews and metal analysis protocols
RCT: Randomised controlled trial
ROM: Range of motion
TDR: Total disc replacement
VAS: Visual analogue scale
